# High burden of antimicrobial resistance among gram negative bacteria causing healthcare associated infections in a critical care unit of Nepal

**DOI:** 10.1186/s13756-017-0222-z

**Published:** 2017-06-15

**Authors:** Narayan Prasad Parajuli, Subhash Prasad Acharya, Shyam Kumar Mishra, Keshab Parajuli, Basista Prasad Rijal, Bharat Mani Pokhrel

**Affiliations:** 1 Department of Clinical Microbiology, Institute of Medicine, Tribhuvan University Teaching Hospital, Kathmandu, Nepal; 2 Department of Laboratory Medicine, Manmohan Memorial Institute of Health Sciences, Khola Pravesh Marg, Soalteemode, P.O. Box No. 15201, Kathmandu, Nepal; 3 Department of Anesthesiology and Critical Care, Institute of Medicine, Tribhuvan University Teaching Hospital, Kathmandu, Nepal

**Keywords:** Healthcare associated infections, ICU, MDR bacteria, β-lactamases, Nepal

## Abstract

**Background:**

Healthcare associated infections (HCAI) and antimicrobial resistance are principal threats to the patients of intensive care units and are the major determining factors for patient outcome. They are associated with increased morbidity, mortality, excess hospitalization and financial costs. The present study is an attempt to investigate the spectrum and antimicrobial resistance of bacterial isolates involved in healthcare associated infections (HCAI) in the patients of a critical care unit at a tertiary care university hospital in Kathmandu, Nepal.

**Methods:**

A laboratory based study was conducted over the period of 15 months (January 2014 to March 2015) among the patients of intensive care unit of Tribhuvan University Teaching Hospital, Kathmandu, Nepal. Clinical specimens from patients with suspected healthcare-associated infection were processed and bacterial isolates were identified with standard microbiological methods. Antimicrobial susceptibilities of the isolated strains were determined according to the CLSI guidelines and β-lactamases (ESBL, AmpC, MBL and KPC) were detected by various phenotypic tests.

**Results:**

One hundred and forty nine clinical specimens received from 135 patients suspected of HCAI (out of 491 patients) were found with significant bacterial growth. Specimens were from patients suspected of hospital-acquired pneumonia (16%, 79/491), bloodstream infections (5.7%, 28/491), surgical site infections (4.7%, 23/491), and urinary tract infections (3.9%, 19/491). *Acinetobacter* spp., *Klebsiella* spp., *Escherichia coli* and *Burkholderia cepacia* were the leading bacterial pathogens. Extremely high level of drug resistance (95.8%) along with the production of β-lactamases (ESBL; 43.7%, AmpC; 27.5%), MBL; 50.2% and KPC; 4.2%) was observed among Gram negative bacterial isolates.

**Conclusion:**

Healthcare associated infections are very common in our ICU. Gram negative bacterial pathogens are major culprits associated with these infections and there is alarming state of drug resistance among these isolates. Continuous surveillance and establishment of preventive and control measures of healthcare associated infections are urgently needed in our setting.

## Background

Infection is the most common presentation among hospitalized patients of intensive care unit (ICU), and in many instances, is a determining factor for patient outcomes [1, 2]. Healthcare associated infections (HCAIs), in particular, are the major risks associated with critically ill patients of ICU, due to the reduced host defenses, frequent use of invasive medical devices, administration of multiple drugs, cross transmission of pathogens among patients and staffs, and inadequate infection control procedures [3, 4]. Hence, intensive care units (ICUs) are now often recognised as the epicenter of infections in the hospital [5]. Pneumonia, surgical-site infections, catheter-related bloodstream infections and urinary tract infections are currently the most common cause of death in ICU due to healthcare associated infections [6]. According to a large surveillance study, more than 70% of critically ill patients receive an antimicrobial drug during their ICU stay either for prophylaxis or for therapy [1]. Nevertheless, in the recent years, therapeutic drugs are being progressively ineffective against bacterial infections, threatning the success of routine treatment [7]. The major consequences of this problem are increased patient morbidity, mortality, health care related expenses and treatment failure [8, 9].

β-lactam antibiotics are the major bulk of prescribed antibiotics in ICUs across the globe because of their efficacy, broad spectra and low toxicity [10]. However, irrational use of these antibiotics has resulted in the development and spread of drug resistant bacterial pathogens especially in the developing countries [11]. Of the particular concern, increased occurrence of Gram negative bacteria, including multidrug resistant nonfermenters (*Acinetobacter baumannii and Pseudomonas* species) and Enterobacteriaceae producing extended-spectrum beta-lactamase(ESBL) and carbapenemases in severe healthcare-associated infections has evolved as a significant clinical threat for medical fraternity in the recent decades [12, 13]. The broad substrate profile of these enzymes may affect entire beta lactam agents, and also the organisms with these enzymes are additionally found resistant to aminoglycosides and fluoroquinolones, further compromising the therapeutic choices for severe infections in ICU [14, 15].

In Nepal, international guidelines on initial antibiotic selection are generally applied in ICUs and empiric choices are made for serious ICU related infections. Thorough knowledge of epidemiology, spectrum and nature of infections along with susceptibilities of causative organisms are extremely valuable for empirical treatment of severe infections in intensive care hospital settings. Therefore, it would be an effective measure for policy formulation of judicious antimicrobial therapy for critically ill patients in the intensive care units of various hospitals in our country. In this perspective, we aimed to determine the incidence of multidrug resistant bacteria, their susceptibilities and common mechanisms of drug resistance involved in healthcare associated infections in the critically ill patients of an intensive care unit at a tertiary care teaching hospital in Kathmandu, Nepal.

## Methods

A laboratory based study was conducted at the department of clinical microbiology of the Tribhuvan University Teaching Hospital in Kathmandu, Nepal from January 2014 to March 2015 (over a period of 15 months). All clinical specimens from the patients suspected of healthcare associated infections (after 48 h of admission to the ICU) representing various body systems viz. blood, urine, endotracheal aspirate (ETA), bronchoalveolar lavage fluid (BAL), pleural fluid, pus, peritoneal fluid, cerebrospinal fluid (CSF), CSF shunt, central venous catheter (CVC) etc. were included in the study. The specimens were collected appropriately by trained ICU nurses and transported to the clinical microbiology laboratory with minimal time delay. Specimens that strictly met the criteria recommended by the American Society for Microbiology (ASM) [16] were selected for further processing and analysis. However, specimens not fulfilling the ASM criteria and duplicate specimens from the same patient were excluded.

### Inoculation of the specimen and identification of the isolates

The clinical specimens were inoculated onto suitable culture medium according to their specific requirements. Respiratory specimens and CSF samples were cultured on Chocolate agar (CHA), 5% Sheep Blood Agar (BA) and MacConkey Agar (MA) (HiMedia, Mumbai, India) plates. Blood specimens were first enriched with Brain Heart Infusion broth (BHI) and then subcultured on to the 5% Sheep Blood Agar (BA) and MacConkey Agar (MA) (HiMedia, Mumbai, India) plates. The vascular catheter tips (CVC tips) were inoculated onto 5% Sheep blood Agar (BA), MacConkey agar (MA) (HiMedia Mumbai, India) according to Maki’s roll plate method [17] by semiquantitative technique. Similarly, surgical specimens, wound swab, pus and urine specimens were plated onto 5% Sheep Blood Agar (BA) and MacConkey Agar (MA) (HiMedia, Mumbai, India) plates. The CHA plates were incubated in a CO_2_ incubator (10% CO_2_) at 37 °C for 24 h. The BA and MA plates were incubated at 37 °C for 24 h in an aerobic atmosphere. Identification of significant isolates that are associated with healthcare associated infections was performed following standard microbiological techniques which involved morphological appearance of the colonies; Gram’s staining, catalase test, coagulase test, oxidase test with other biochemical parameters [16]. Assurance of pure culture inoculum was done by setting purity plate along with the biochemical tests.

### Antibiotic susceptibility testing

The susceptibility of bacterial isolates against different antibiotics was determined by the disk diffusion method [modified Kirby-Bauer method] on Mueller Hinton agar (Hi-Media, India) following standard procedures recommended by the Clinical and Laboratory Standards Institute (CLSI), Wayne, USA [18]. For this purpose following antibiotics with specified concentrations were used; ampicillin (10 μg), ampicillin-sulbactam (10/10 μg), ceftazidime (30 μg), ceftriaxone (30 μg), cefepime (30 μg), cefoxitin (30 μg), piperacillin-tazobactam (100/10 μg, aztreonam (30 μg), imipenem (10 μg), meropenem (10 μg), gentamycin (10 μg), amikacin (30 μg), ciprofloxacin (5 μg), ofloxacin (5 μg), levofloxacin (5 μg), trimethoprim-sulphamethoxazole/co-trimoxazole (25 μg), polymixin B (300unit), colistin sulphate (10 μg)] and tigecycline (30 μg) from HiMedia Laboratories, India. Interpretations of antibiotic susceptibility results were made according to the guidelines of interpretative zone diameters of CLSI [18]. *Escherichia coli* ATCC 25922 and *Pseudomonas aeruginosa* ATCC 27853 were used as the control organisms for antibiotic sensitivity.

### Identification of Multidrug Resistant (MDR), Extensively Drug Resistant (XDR) and Pan Drug Resistance (PDR) isolates

Multidrug resistant (MDR) bacterial isolates were identified according to the criteria recommended by international expert committee of the European Centre for Disease Prevention and Control (ECDC) and the Centers for Disease Control and Prevention (CDC) [19]. In this study, the isolate resistant to at least one antimicrobial from three different group of first line drugs tested was regarded as multidrug resistant (MDR). Extensively drug resistant (XDR) isolates were identified when the isolates were resistant to at least one agent in all but two or fewer antimicrobial categories (i.e. bacterial isolates remain susceptible to only one or two categories). Pan drug resistant (PDR) was defined as non-susceptibility to all agents in all antimicrobial categories (i.e. no agents tested were susceptible for that organism) [19].

### Detection of ESBL, MBL, AmpC and KPC β-lactamases

#### Testing of extended spectrum β-lactamase (ESBL) producers

The initial screening test for the ESBL production was performed by using one of three antibiotics; ceftriaxone (CRO 30 μg), ceftazidime (CAZ 30 μg) or cefotaxime (CTX 30 μg) disks (HiMedia, Mumbai, India). If the zone of inhibition (ZOI) was ≤25 mm for CRO,≤22 mm for CAZ and/or ≤ 27 mm for CTX, the isolate was considered a potential ESBL producer as recommended by CLSI [18]. Isolates that were suspected as ESBL-producer by screen test were tested further by combination disk test (CDT). In this test, Ceftazidime (30 μg) disks alone and in combination with clavulanic acid (ceftazidime + clavulanic Acid, 30/10 μg) disks, were applied onto a plate of Mueller Hinton Agar (MHA) which was inoculated with the test strain and then incubated in ambient air for 16-18 h of incubation at 35 ± 2 °C. Isolate that showed increase of ≥5 mm in the zone of inhibition of the combination discs in comparison to that of the ceftazidime disk alone was considered an ESBL producer [18].

### Testing of metallo β-lactamase (MBL) producers

Isolates that were found non-susceptible to third generation cephalosporins (ceftazidime), imipenem or meropenem in Kirby Bauer disk diffusion method were presumptively considered MBL producers and were confirmed by the imipenem disk with ethylene diamine tetra acetic acid (EDTA) method. Briefly, the test inoculums (comparable to 0.5 McFarland standards) were prepared and transferred on to Mueller Hinton agar plates. In the combination disk test for MBL, two imipenem (IPM) disks (10 μg), a plain imipenem disk and another containing 10 μl of 0.1 M (292 μg) anhydrous EDTA (Sigma Chemicals, St. Louis, MO), were placed 20 mm apart. An increase in the zone size of more than or equal to 7 mm for imipenem-EDTA disk compared to imipenem disk alone indicated MBL producer strain as described by Yong et al. [20].

### Testing of AmpC β-lactamase producers

Screening of AmpC β-lactamase production was carried out by cefoxitin disk. Isolates that yielded a zone diameter less than 18 mm (screen positive) were further subjected to confirmatory testing. Cefoxitin (30 μg) disk alone and cefoxitin (30 μg) disk containing 10 μl of 300 μg/ml PBA (Phenyl Boronic Acid) were placed at 20 mm distance. An increase in zone of inhibition by at least 5 mm around Cefoxitin disk containing Boronic acid after overnight incubation at 37 °C were considered as positive for AmpC production by the isolates [21].

### Testing of *Klebsiella pneumoniae* carbapenemase (KPC) producers

The isolates that tested for MBL detection were also subjected to KPC production. Phenotypic test for KPC detection was performed by combination disk method. In this test, meropenem (MEM) (10 μg) disk alone and a meropenem disk containing 10 μl (300 μg/ml) 3-AminoPhenyl Boronic Acid (3-APBA) (Tokyo Chemical Co. Ltd., Japan) were placed 20 mm apart centre to centre. An increase in zone diameter of more than 5 mm around the MEM- PBA disk compared to that of the MEM disk alone was considered positive for KPC. To exclude the AmpC overproduction in the same isolate, four disk test for differentiation of carbapenemeses described by Tsakris et al. was used [22].

### Ethical consideration

Written approval (Ref No: 129(6-11-E)/2070/71) was obtained from Institutional Review Board (IRB) of Institute of Medicine after submitting and presenting research proposal. In addition, written consent was taken from every patient or their guardian for participation into this study before enrollment.

### Data processing and analysis

Data regarding patient demographics, HCAI types, bacterial isolates, antimicrobial susceptibilities and resistance determinants were entered in to a computer program. Data were analyzed using SPSS 20.0 version and interpreted according to frequency distribution, percentage.

## Results

### Patient demographics

During the study period, a total of 568 patients were admitted to the intensive care unit of our hospital. Forty six patients were transferred to another unit, 17 died and 14 patients left against medical advice within 48 h of their admission. Among 491 patients stayed more than 48 h in the ICU, male patients (280/491, 57%) were more than their female counterparts (211/491, 43%). The median age of patient was 41.4 years (IQR; 35-60) and maximum numbers of patients were from the age group 46-55 years.

### Distribution of specimens by suspected HCAI

After clinico-bacteriological analysis, 149 clinical specimens received from 135 patients (out of 491 patients admitted to the ICU) suspected of HCAI were found to be associated with significant bacterial growth. Among specimens with significant growth, majority were from suspected hospital acquired pneumonia [(53.0%, 79/149), (overall; 16.0%, 79/491)] followed by bloodstream infections [(18.8%, 28/149), (overall; 5.7%, 28/491)], surgical site infections [(15.4%, 23/149), overall (4.7%, 23/491)] and urinary tract infections (12.8%,19/149), overall (3.9%, 19/491)] (Table 1).

**Table 1:** Distribution of specimens by suspected Healthcare Associated Infections (HCAI)

Type of Infections	No.	%	Overall incidence %
Hospital acquired pneumonia	79	53.0	16.0
Bloodstream infections	28	18.8	5.7
Surgical site infections	23	15.4	4.7
Urinary tract infections	19	12.8	3.9
Total	149	100.0	27.4

### Distribution of bacterial isolates

Diverse bacterial etiology was noted among HCAIs (Table 2). *Acinetobacter* spp*.* (51, 34.9%) was the leading organism followed by *Klebsiella* spp. (37, 25.3%), *Escherichia coli* (31, 21.2%), and *Pseudomonas* spp. (24, 16.4%). Hospital acquired pneumonia were predominantly associated with *Acinetobacter* spp. (38.1%) and *Klebsiella* spp. (21.6%) while bloodstream infections were caused by *Burkholderia cepacia* (47.3%), surgical site infections caused by *Pseudomonas* spp. (31.0%) and urinary tract infections caused by *Klebsiella* spp. (42.8%). Among total 28 bloodstream infections, 19 were associated with Gram negative bacteria and remaining 9 were Gram positive bacteria (data not presented).

**Table 2:** Distribution of Bacterial isolates

Bacterial isolates	Total number (%)			
	HAP(*n* = 79)	BSI(*n* = 28)	SSI (*n* = 23)	UTI (*n* = 19)
*Acinetobacter* spp. (*n* = 51)	37(38.1)	3(15.7)	7(24.1)	4(19.0)
*Klebsiella spp.* (*n* = 37)	21(21.6)	1(5.2)	6(20.6)	9(42.8)
*Escherichia coli* (*n* = 31)	19(19.5)	5(26.3)	2(6.9)	5(23.9)
*Pseudomonas spp.*(*n* = 24)	12(12.4)	0(0.0)	9(31.0)	3(14.3)
*Burkholderia cepacia* (*n* = 14)	6(6.2)	9(47.3)	0(0.0)	0(0.0)
*Citrobacter spp.* (*n* = 8)	2(2.0)	1(5.2)	5(17.2)	0(0.0)
Total isolates (*n* = 165)	97(100.0)	19(100.0)	29(100.0)	21(100.0)

### Antimicrobial susceptibility pattern of isolates

#### Antibiogram of nonfermenters

Gram negative nonfermenters were variably resistant to tested antimicrobials (Table 3). Entire isolates of *Acinetobacter* and *Burkholderia* spp. were resistant to cephalosporins, while almost 92% of *Pseudomonas* spp. were cephalosporin resistant. Resistant to fluoroquinolones was observed higher (upto100%) in *Burkholderia* spp. when compared to that in *Acinetobacter* spp. (94.2%) and *Pseudomonas* spp. (95.8%). Carbapenems, the preferred regimens in ICU, were highly resistant in *Acinetobacter* spp. (upto86.4%) and *Pseudomonas* spp. (62.5%) but were effective against *Burkholderia* spp. (20% resistance).

**Table 3:** Antibiogram of Nonfermenters (*n* = 90)

Antibiotics	% Resistance among bacterial isolates		
	*Acinetobacter* spp. (*n* = 51)	*P. aeruginosa* (*n* = 24)	*Burkholderia* spp. (*n* = 15)
Cotrimoxazole	100	-	0
Ceftazidime	100	91.6	100
Cefepime	100	91.6	100
Ciprofloxacin	94.2	95.8	100
Levofloxacin	76.5	87.5	93.3
Gentamycin	92.2	62.5	86.6
Amikacin	84.4	37.5	67
Piperacillin Tazobactam	92.2	67	60
Ampicillin Sulbactam	92.2	-	-
Cefoperazone Sulbactam	90.2	67	86.6
Imipenem	86.4	62.5	20
Meropenem	84.4	62.5	20
Polymixin B	0	0	–
Colistin Sulphate	0	0	–
Tigecycline	0	–	–

### Antibiogram of Enterobacterial isolates

In this study, entire isolates of enterobacteriaceae were resistant to third generation cephalosporins. *Klebsiella* spp. were resistant to ciprofloxacin (86.4%), gentamycin (83.7%), piperacillin tazobactam (81.0%) and imipenem (48.6%). Almost similar rates of resistance were observed in *Escherichia coli,* except for carbapenems (19.3% resistance). Although, the number of *Citrobacter* spp. was small, majority of them were resistant to cephalosporins, fluoroquinolones and aminoglycosides (Table 4).

**Table 4:** Antibiogram of Enterobacterial isolates (*n* = 76)

Antibiotics	% Resistance among bacterial isolates		
	*Escherichia coli* (*n* = 31)	*Klebsiella* spp. (*n* = 37)	*Citrobacter* spp. (*n* = 8)
Ampicillin	100	–	–
Cotrimoxazole	64.5	100	100
Cefotaxime	100	100	100
Cefepime	100	100	100
Ciprofloxacin	90.3	86.4	100
Levofloxacin	77.4	72.9	100
Gentamycin	64.5	83.7	100
Amikacin	45.1	83.7	100
Piperacillin Tazobactam	48.3	81.0	87.5
Cefoperazone Sulbactam	48.3	83.7	87.5
Imipenem	19.3	48.6	25
Meropenem	19.3	48.6	25
Polymixin B	0	0	0
Colistin Sulphate	0	0	0
Tigecycline	0	0	0

### MDR, XDR and β-lactamase producing bacterial isolates

Nearly 96% of the Gram negative bacterial isolates causing nosocomial infections were found multidrug resistant and 43.3% isolates were extensively drug resistant (XDR). In this study, rates of beta lactamase producing bacterial isolates was extremely high (ESBL; 43.7%, AmpC; 27.5%), MBL; 50.2% and KPC; 4.2%). *Escherichia coli* was major ESBL producer (70.9%) followed by *Citrobacter* spp. (62.5%) and *Klebsiella* spp. (59.4%). Major MBL enzyme producers were *Acinetobacter* spp. (78.8%), *Pseudomonas* spp. (62.5%) and *Klebsiella* spp. (48.6%). Small proportion of *Klebsiella* (10.8%) and *Acinetobacter* spp. (5.7%) were KPC producers (Table 5).

**Table 5:** MDR,XDR and β-lactamse producing bacterial isolates

Bacterial isolates	% MDR	% XDR	β- lactamases producers			
			% ESBL	% MBL	% AmpC	% KPC
*Escherichia coli*	100	19.3	70.9	16.1	38.7	0
*Klebsiella spp.*	100	48.6	59.4	48.6	32.4	10.8
*Acinetobacter spp.*	94.2	84.4	15.4	78.8	42.3	5.7
*Pseudomonas spp.*	83.3	62.5	33.4	62.5	–	0
*Burkholderia spp.*	100	20	53.4	20	–	0
*Citrobacter spp.*	100	25	62.5	25	–	0
Total	95.8	43.3	43.7	50.2	27.5	4.2

## Discussion

Increased consumption of antimicrobial regimens, higher prevalence and dissemination of drug resistance among nosocomial pathogens and poor infection control strategies for prevention of healthcare associated infections are the rising problems in Nepalese hospitals [23]. The problem is several-folds high in the intensive care units where collection of severely ill patients from all over the hospital units with varieties of pathological profile and etiological agents exists. Therefore, identification of the underlying pattern of drug resistance among microorganisms in every hospital is the key to success in the appropriate treatment of patients. This issue is of interest especially in Nepalese ICUs where the highest prevalence of patients on antibiotic treatment is frequently reported.

The rate of healthcare associated infections varies globally and higher rates have been reported from developing countries [24]. Moreover, the type of hospital setting (ward or intensive care unit), patient population and the precise definition and surveillance techniques used to identify the healthcare associated infections are responsible for variable incidences [25]. Healthcare associated infections are frequent in our study as previously described by Sah et al. from same hospital [26], although they have included the patients from all sections of hospital, providing heterogeneity of the cases. However, extremely high incidences have been documented in recent reports from India (11.9-17.7%) [27, 28]. In the well known EPIC II study by Vincent et al., 51% of the patients in ICU were found infected [1]. Another multicentre cohort study yielded the rate of infections in ICU patients around the globe to be ranging from 2.3 to 49.3% [29]. However, the higher rates in multicentre studies are due to the heterogeneous patients, study parameters, voluntary participation and reporting, as well as the variation in surveillance techniques [30].

We observed hospital acquired pneumonia (53%) to be the most common healthcare associated infection in this study, followed by bloodstream infections (18.8%), surgical site infections (15.4%) and urinary tract infections (12.8%) which are comparable to the findings of large EPIC II study [1]. Similar findings were also been reported from epidemiological study of infections in ICU population by Cosic et al. from Brazil [31] and Dasgupta et al. from India [28]. However, in a recent report, Mythri and Kashinath pointed UTI as the most common infection, followed by pneumonia and surgical site infections [27]. The site distribution of infections in various studies might be attributable to the type of ICUs and patient population. Our report broadly corroborates to the findings of earlier studies mentioned above.

Non fermentative Gram negative bacteria including enterobacteriaceae are increasingly reported as the cause of healthcare associated infections worldwide [32]. *Acinetobacter* species, major pathogenic organism in health-care-associated infections, was found to be the most common organisms in our ICU. Majority of the infections associated with *Acinetobacter* were pneumonia and surgical site infections. Similar spectrum of bacterial pathogens in healthcare associated infections in ICU has been documented by other studies too [26, 33]. However, *Burkholderia cepacia* was the most common bacteria isolated from bloodstream infections in our ICU. Increased use of pharmaceutical derivatives and accumulation of sicker patients with variable infections might be the reason for our shifting etiology.

Antimicrobial resistance is a recognized problem in South Asian region with high levels of resistance among Gram negative organisms reported frequently [34]. It is well known fact that resistance is due to extreme antimicrobial consumption [11], and overuse of antibiotics can be surmised as one of the factors contributing to the high rates of antimicrobial resistance in Nepal. In this study, many microorganisms found as resistant to different antimicrobial agents and in some cases to nearly all agents representing an alarming scenario in our intensive care setting. Nearly 96% of the Gram negative bacterial isolates causing nosocomial infections were found multidrug resistant, which is highest ever rate of MDR bacteria reported from our country. Earlier reports from Nepal have reported upto 95% of the nosocomial isolates as drug resistant [35, 36]. Further in this study, not only the isolates were multidrug resistant, a significant proportion (43.3%) of our isolates was extensively drug resistant (XDR). The high level of drug resistance in Gram negative isolates have been described as a result of the production of different 훽-lactamases, multiple efflux pumps, decreased drug uptake, and other drug modifying enzymes [37].

Gram negative non-fermenters were more resistant to antimicrobial drugs in this study. *Acinetobacter* isolates were found highly resistant to carbapenem (86.4%), aminoglycosides (93%) and cephalosporins (100%) groups of antibiotics which is nearly twofold high than that reported by Mishra et al. from same hospital (nearly 89% resistance in cephalosporins and 50% in carbapenem) [35]. However, the resistance rate of Gram negative nonfermenters in our study is comparable to the study of Xia et al. from China [38] and Fatima et al. from Pakistan [39]. Moreover, a SENTRY study also reported that Gram negative bacterial resistance to imipenem changed from 34.5% in 2006 to 59.8% in 2009 across the world [40]. In addition to this, the increasing emergence of highly aminoglycosides-resistant strains is also cause of major concern. Our result on amikacin resistance (up to 85%) is higher than that (54%) of previous study from same hospital [35]. Although none of the *Acinetobacter* strains in our study was tigecycline and colistin resistant, it has been indicated as an emerging therapeutic problem which may severely compromise the treatment of MDR *Acinetobacter* spp. infections [41]. This high prevalence of multi drug resistance in *Acinetobacter spp.* may be due to high chance of acquisition of resistance gene and their ability to persist and multiply in hospital environment [42]. On the other hand, *Pseudomonas aeruginosa* and *Burkholderia cepacia* are intrinsically resistant to several antibiotics because of the low permeability of their outer-membrane, the constitutive expression of various efflux pumps, and the production of antibiotic-inactivating enzymes (e.g., cephalosporinases) [43].

*Enterobacteriaceae*, including *Escherichia coli, Klebsiella* spp. and *Citrobacter* spp. were also found resistant to multiple antibiotics. Resistance of enterobacterial strains towards cephalosporins (100%), fluoroquinolones (86.4 to 100%), aminoglycosides (45.1 to 100%) and carbapenems (19.3 to 48.6%) was considerably high when compared to the reported rates from previous studies [36, 44]. However, tigecycline and polymixins showed excellent effect against MDR Gram-negative enterobacterial isolates. High antibiotic resistance rate against commonly used antibiotics is a disadvantage for health care system in countries like Nepal as it can greatly affect patient management especially in critical care units.

Beta lactamases are the hydrolytic enzymes thwarting the functional part of *β*-lactam antibiotics used for the treatment of most bacterial infections. The menacing state of resistance in Gram negative isolates towards the cephalosporins, carbapenems and other antibiotics could be attributed to ESBL, AmpC β-lactamase, carbapenamase producers and some other relevant underlying mechanisms [45]. In this study, the rates of beta lactamase production, including ESBL, AmpC, MBL, and KPC were alarmingly high. *Escherichia coli, Klebsiella* spp*.*, *Citrobacter* spp., *Pseudomonas* spp. and *Burkholderia* spp. were major ESBL producers. Previously, Shrestha et al. in 2011 have also reported 28.57% of *Escherichia coli*, 8.33% of *Klebsiella spp.* and 2.38% of *Pseudomonas aeruginosa* were ESBL producers [46]. However, in an Indian study, the rate of ESBL production among nosocomial isolates was 50% in *Escherichia coli* and 67% in *Klebsiella pneumoniae* respectively [41]. Variations in ESBL rates may be ascribed to antibiotic prescribing habits and endemicity of pathogens harboring the genes for ESBL production. Furthermore, we detect 27.5% AmpC producing isolates, including *Acinetobacter spp.* (42.3%) followed by *Escherichia coli* (38.7%) and *Klebsiella pneumoniae* (32.4%) which is comparative to the previous reports of Baral et al. [47] and Khanal et al. [36] from nearby hospital. However, in an Indian study lower rate (14.2%) of AmpC producing isolates were documented [48].

High resistance of carbapenems in our study has been supported by the detection of carbapenemase enzyme in the bacterial isolates. Major bacterial isolates producing MBL were *Acinetobacter* spp., *Pseudomonas* spp., *Klebsiella* spp. and *Burkholderia* spp. Although, till date, there is no single article regarding detection of KPC from Nepal, this study attempted to find out the KPC producing bacterial isolates in ICU population. In Nepal, carbapenamase producing bacteria are poorly reported. Mishra et al. documented the rate of MBL producers to be 1.3% including *Acinetobacter* (4.3%), *Pseudomonas* (3.3%) [49], but in a study by Shrestha et al. the rate of MBL was 17.43% among nosocomial isolates including *Acinetobacter* (47.22%), *Pseudomonas* (2.38%) and of *Klebsiella* spp. (4.17%) [46]. The emergence of carbapenamase in Gram negative bacilli is becoming a therapeutic challenge as these enzymes possess high hydrolytic activity that leads to degradation of higher generation cephalosporins and carbapenems. Furthermore, these plasmid mediated genes have the ability spread rapidly to other species of Gram negative bacilli [50]. Therefore rapid detection of these resistance determinants is necessary to modify therapy and to initiate effective infection control to prevent their dissemination.

### Limitations

We could not evaluate the risk factors and outcomes of nosocomial infections associated with drug resistant bacteria. Due to the unavailability of sufficient data from the patients without healthcare associated infections, statistical comparison between the patients with and without healthcare associated infections could not been made. Further, it was limited to a mixed adult ICU of a teaching hospital leaving other surgical and pediatric ICU patients. Furthermore, molecular characterization of the resistant phenotypes and their epidemiology would be more significant in public health perspective.

## Conclusion

From this study, it becomes clear that healthcare associated infections caused by drug resistant bacteria are major problems in our ICU. Menacing state of drug resistance among Gram negative pathogens associated with these infections is particularly worrisome. It is very important to control this situation before it takes a deadly shape. Established recommendations including adequately identifying the pathogen, choosing correct antibiotics, limiting their excess use, improving resistance surveillance systems will help controlling this situation.

## Abbreviations

ASM: American Society for Microbiology
BSI: Bloodstream infection
CLSI: Clinical and Laboratory Standards Institute
ESBL: Extended spectrum β-lactamases
HAP: Hospital acquired pneumonia
HCAI: Healthcare associated infection
ICU: Intensive care unit
KPC: Klebsiella pneumoniae carbapenamase
MBL: Metallo β-lactamases
MDR: Multidrug resistant
SSI: Surgical site infection
UTI: Urinary tract infection
XDR: Extensively drug resistant
